# Monoclonal antibody therapy in patients with severe asthma and COPD: rethinking treatment pathways and success

**DOI:** 10.1136/bmjresp-2026-004232

**Published:** 2026-07-31

**Authors:** Rachel Burton, Maria Puthoor, Fred Fyles, Lisa Lewis, Hannah Joplin, Hassan Burhan

**Affiliations:** 1 Institute of Life Course & Medical Sciences, University of Liverpool, Liverpool, England, UK; 2 Liverpool University Hospitals NHS Foundation Trust, Liverpool, England, UK; 3 Clinical Sciences, Liverpool School of Tropical Medicine, Liverpool, England, UK; 4 Institute of Infection, Veterinary and Ecological Sciences, University of Liverpool, Liverpool, UK

**Keywords:** Asthma, Asthma Guidelines, Asthma Pharmacology, COPD Exacerbations, COPD Pharmacology, Pulmonary Disease, Chronic Obstructive

## Abstract

The new National Institute for Health and Care Excellence (NICE) guidance on the use of dupilumab in chronic obstructive pulmonary disease (COPD) recommends using dupilumab as an add-on therapy for patients with uncontrolled COPD and raised blood eosinophils who either have one severe exacerbation or two moderate exacerbations despite triple therapy. This contrasts with the guidance for commencing and continuing monoclonal antibody therapy (MAb) in asthma and will have significant implications for severe asthma services caring for patients with coexisting asthma and COPD or those with asthma and a significant smoking history. Patients with asthma and COPD often experience poorer outcomes than patients with asthma or COPD alone. They are traditionally managed according to asthma pharmacotherapy guidelines; however, under the new guidance this may change. Our perspective is supported by local audit data helping us to reflect on our current practice as a large tertiary severe asthma service of initiating MAb in patients with coexisting asthma and COPD.

Disparities in eligibility and access to dupilumab in asthma and COPD guidelines expose key challenges about the best clinical setting for managing patients with coexisting disease, the need for distinct treatment pathways and personalised definitions of treatment success, and whether the current positioning of dupilumab as a second-line therapy within severe asthma pathways in England remains appropriate. Addressing these issues will be critical to supporting equitable clinically appropriate access to monoclonal antibodies in patients with coexisting asthma and COPD in the future.

 The National Institute for Health and Care Excellence (NICE) has recently published guidance on the use of dupilumab in chronic obstructive pulmonary disease (COPD), representing a significant shift in management and carrying important implications for severe asthma services caring for patients with coexisting asthma and COPD.^1^

Patients with both asthma and COPD often experience a high symptom burden, frequent exacerbations and a poorer quality of life compared with those with asthma or COPD alone.^2–4^

Treatment escalation for patients with coexisting disease has, prior to monoclonal antibody therapy (MAb) availability for COPD, followed asthma pharmacotherapy pathways.^5^ If patients exacerbate despite adherence to maximal inhaled therapy, they are usually referred to a severe asthma multidisciplinary team for consideration of a MAb under the asthma indication.

MAbs are well established in severe asthma and are now being evaluated in COPD, with dupilumab recently licensed for select COPD patients.^6^ New NICE guidelines propose using dupilumab as an add-on therapy for patients with uncontrolled COPD and raised blood eosinophils (defined as ≥0.3 x 10^9^ cells per litre) who either have one severe exacerbation or two moderate exacerbations despite triple therapy (or dual therapy if inhaled corticosteroids (ICS) are not appropriate) in the last 12 months.^1^ This compares to severe asthma, where patients require 3–4 exacerbations, with no differentiation in severity, to be eligible for a MAb.^7–10^

Phase III clinical trial data suggest although MAb therapy in COPD may reduce exacerbation frequency, the magnitude of benefit is generally smaller than that observed in asthma.^11 12^

In asthma, MAb success is typically defined as a >50% reduction in exacerbations requiring oral prednisolone (OCS, oral corticosteroids) and/or a reduction in maintenance prednisolone at 12 months.^7–10^ In comparison, the NICE guidelines propose only stopping dupilumab in COPD if the number of severe exacerbations is higher or the number of severe exacerbations is the same but the number of moderate exacerbations is increased.^1^

However, for patients with coexisting asthma and COPD, uncertainties remain regarding optimal management, particularly around the use of MAbs and how treatment success should be defined following initiation. This challenge may be amplified given the substantial differences in both eligibility criteria and outcome measures used to assess treatment response in asthma compared with COPD and may be relevant when considering MAb switching following either partial response or failure.

## Our experience

To understand the potential implications of these recommendations on our current practice, we retrospectively reviewed our clinical experience of managing patients with coexisting asthma and COPD treated with a MAb for asthma. This reflection is based on patients commenced on a MAb between January 2022 and December 2023 at a single, large, tertiary asthma service in England. These data were collected as part of a locally registered and approved service evaluation.

To qualify for a MAb, under the asthma indication in England at that time, patients required:^7–10^

- A confirmed diagnosis of asthma, with either reversibility (an improvement of 200 mL and 12% in forced expiratory volume in 1 s (FEV1) following a bronchodilator), evidence of bronchial hyper-reactivity or significant variability in FEV1 over time (defined as 200 mL and 12% difference in FEV1 between clinic appointments).
- Adequate adherence and good inhaler technique with high-dose ICS and long-acting beta agonist (LABA) treatment.
- Evidence of frequent OCS or requirement of maintenance prednisolone for the management of their respiratory disease despite optimised inhaled therapy.
- Prior to tezepelumab availability, patients required documented evidence of type 2 inflammation in the preceding 12 months.

For the purpose of this service evaluation, patients were considered to have both asthma and COPD if they also had a physician diagnosis of COPD, obstructive spirometry post-bronchodilator (defined as an FEV1/forced vital capacity ratio <70%) and a significant smoking history (>10 pack year history).

Baseline clinical characteristics and 12 month follow-up data were collected from electronic patient notes.

Between January 2022 and December 2023, 375 patients were commenced on a MAb for asthma, of which 23 (23/375, 6%) had a concurrent COPD diagnosis. Median age was 69 years (IQR 62–75) and most were female (61%, 14/23). Almost all patients were ex-smokers (87%, 20/23), reflecting current local practice in which engagement with smoking cessation services is strongly encouraged and typically required prior to MAb approval. The median pack-year history was 30 years (IQR 20–42 pack years).

Half of patients (12/23, 52%) had significant reversibility (defined as more than 200 mL and 12%) to support their asthma diagnosis. Three-quarters (17/23, 74%) had radiographic evidence of emphysema on CT scan. Almost all patients (22/23, 96%) were on a minimum of triple therapy with a high dose ICS, LABA and long-acting muscarinic antagonist prior to starting a MAb. Median number of controllers was 4 (IQR 4–5).

Baseline median annualised exacerbation frequency (defined as exacerbations requiring high dose OCS) prior to starting was 6 (IQR 4–7). Due to MAb eligibility requirements for severe asthma, only exacerbations requiring OCS are routinely documented and moderate exacerbations requiring antibiotics alone were not collected for this cohort.

Patients were symptomatic (median asthma control questionnaire, ACQ-6, 4.0, IQR 3.6–5.1) and had reduced lung function (median FEV1 per cent predicted 47%, IQR 44%–57% and gas transfer, diffusion capacity of the lung for carbon monoxide, 60% predicted, IQR 47%–66%) at baseline. Chronic airways assessment tool (CAAT score) is not routinely performed in the severe asthma service.

Most patients (19/23, 83%) were MAb naïve. Patients were most commonly started on benralizumab (15/23, 65%), tezepelumab (5/23, 22%) and mepolizumab (3/23, 13%). No patients were commenced on dupilumab as it is unavailable as a first-line MAb for asthma in England.^10^ All those not MAb naïve switched from benralizumab or mepolizumab due to lack of efficacy.

At 12 months, the median ACQ-6 was 3.5 (IQR 2.5–5.1) and the median reduction in OCS was 43% (IQR 11%–71%). Over one-third (39%, 9/23) had a successful trial of treatment (defined as >50% reduction in OCS) and continued with the MAb. A further 17% (4/23) had an extended trial of MAb therapy beyond 12 months due to a borderline response (defined as <50% reduction in OCS, and therefore not meeting the asthma definition of success, but on consultant review demonstrated additional benefit such as cessation of hospital admissions warranting an extension of MAb beyond 12 months under close supervision).

In summary, the cohort of patients with coexisting asthma and COPD commencing a MAb under the asthma indication at our site was relatively small. These data were collected retrospectively, and the population was diverse with significant variation in clinical presentation, risk factors and phenotype which limits the generalisability of our historical experience and clinical practice to other centres.

The number of patients we have previously reviewed likely underestimates the burden of comorbid asthma and COPD in our population and historical practice reflects clinical decision to only commence a MAb if there was a significant asthma component, limited clinical experience with initiating these treatments in a population who also have COPD and an absence of clearly defined outcome measures for treatment success in this cohort.

Despite almost all patients requiring evidence of type 2 inflammation, no patients were commenced on dupilumab. MAb choice was influenced by the fact this review was undertaken during a period in which tezepelumab had only recently been approved for use within the NHS, and dupilumab was only prescribable as a second-line agent for asthma in accordance with the NICE criteria.^10^ Decision to commence either benralizumab, mepolizumab or tezepelumab usually defaults to the presenting clinician preference, considering biomarker activity and other comorbidities such as the presence of nasal disease.

## Conclusion

The number of patients with coexisting asthma and COPD commencing a MAb is likely to increase as emerging evidence supports the use of selected MAbs in COPD. New NICE guidance on dupilumab in COPD highlights disparities in eligibility and access to the same therapy, exposing potential challenges in the management of patients with coexisting asthma and COPD, as well as those with severe asthma and a significant smoking history. Patients with overlapping asthma and COPD can have varying phenotypes, inflammatory profiles and responses to treatment. Within the context of the new COPD guidance, patients with comorbid severe asthma and COPD, although heterogeneous, may benefit from earlier access to a MAb that is efficacious in both conditions, and NICE approved for biologic-naïve patients in COPD but not in severe asthma.

This raises further questions about the best clinical setting for managing this patient cohort, the need for distinct treatment pathways and personalised definitions of treatment success, and whether the current positioning of dupilumab as a second-line therapy within severe asthma pathways in England remains appropriate. Future research to address these issues may be necessary to support equitable, clinically appropriate access to MAbs in patients with coexisting asthma and COPD.
